# Thank you to Microbiome's peer reviewers in 2013

**DOI:** 10.1186/2049-2618-2-2

**Published:** 2014-01-20

**Authors:** Jacques Ravel, K Eric Wommack

**Affiliations:** 1 Institute for Genome Sciences (IGS), University of Maryland, School of Medicine, Department of Microbiology and Immunology, Baltimore, MD, USA; 2 University of Delaware, Delaware Biotechnology Institute, Newark, DE, USA

## Contributing reviewers

We are deeply grateful to all of the names listed below, who have reviewed manuscripts submitted to *Microbiome*. The success of our first year could not have been possible without your help. Looking forward to 2014, we see great things on the horizon. All the 2013 articles were recently listed and will soon be directly accessible through PubMed. This should serve to boost the increases we've seen in the quality and number of submissions to *Microbiome*. We hope that you all will continue to support the journal as a reviewer or, better yet, a contributing author.

Lisa Allen

USA

Florent Angly

Australia

Christophe Benoist

USA

Debby Bogaert

Netherlands

Barbara Campbell

USA

J Gregory Caporaso

USA

Frank Chervenak

USA

Elizabeth Costello

USA

Minucci Daria

Italy

Aaron Darling

Australia

Michael Day

USA

Les Dethlefsen

USA

Suzanne Devkota

USA

Gian Carlo Di Renzo

Italy

Elizabeth Dinsdale

USA

Emiley Eloe-Fadrosh

USA

Larry Forney

USA

Derrick Fouts

USA

James Fox

USA

W. Florian Fricke

USA

Pawel Gajer

USA

Elodie Ghedin

USA

Sean Gibbons

USA

Jack Gilbert

USA

Steven Goodman

USA

Elizabeth Grice

USA

Lindsay Hall

UK

Nathaniel Heintzman

USA

Janet Hill

Canada

Julie Huber

USA

Bonnie Hurwitz

USA

Susan Huse

USA

RE Isaacson

USA

Jacques Izard

USA

Janet Jansson

USA

Rupert Kaul

Canada

Scott Kelley

USA

Dan Knights

USA

Jan Knol

Netherlands

Sarah Lebeer

Belgium

Charles Lee

New Zealand

Huiying Li

USA

Cindy Liu

USA

Bing Ma

USA

Julia Maresca

USA

Helena Mendes-Soares

USA

Emmanuel F. Mongodin

USA

Robin Morgan

USA

Michael Morowitz

USA

Jayne Morrow

USA

Gopinath Balakrish Nair

India

Josef Neu

USA

William Nierman

USA

Nathan Olson

USA

Pinaki Panigrahi

USA

John Parkinson

Canada

Jordan Peccia

USA

Elaine Petrof

USA

Shawn Polson

USA

Christoph Reinhardt

Germany

Kirsti Ritalahti

USA

Jorge Rodrigues

USA

Francisco Rodriguez-Valera

Spain

Forest Rohwer

USA

Jarkko Salojärvi

Finland

Patrick Schloss

Germany

Fergus Shanahan

Ireland

Cody Sheik

USA

Hauke Smidt

Netherlands

Christopher Taylor

USA

Giorgio Trinchieri

USA

Susannah Tringe

USA

Alan Walker

UK

Dana Willner

Australia

Ivan Yap

Malaysia

Shibu Yooseph

USA

Vincent Young

USA

Egija Zaura

Netherlands

